# People with intellectual and multiple disabilities access leisure, communication, and daily activities *via* a new technology-aided program

**DOI:** 10.3389/fpsyg.2022.994416

**Published:** 2022-09-07

**Authors:** Giulio E. Lancioni, Nirbhay N. Singh, Mark F. O’Reilly, Jeff Sigafoos, Gloria Alberti, Alessandra Fiore

**Affiliations:** ^1^Department of Neuroscience and Sense Organs, University of Bari, Bari, Italy; ^2^Department of Psychiatry and Health Behavior, Augusta University, Augusta, GA, United States; ^3^College of Education, University of Texas at Austin, Austin, TX, United States; ^4^School of Education, Victoria University of Wellington, Wellington, New Zealand; ^5^Lega F. D’Oro Research Center, Osimo, Italy

**Keywords:** leisure, communication, daily activities, technology, intellectual disability, sensory impairment, motor impairment

## Abstract

People with mild to moderate intellectual or multiple disabilities may have serious difficulties in accessing leisure events, managing communication exchanges with distant partners, and performing functional daily activities. Recently, efforts were made to develop and assess technology-aided programs aimed at supporting people in all three areas (i.e., leisure, communication, and daily activities). This study assessed a new technology-aided program aimed at helping four participants with intellectual and multiple disabilities in the aforementioned areas. The program, which was implemented following a non-concurrent multiple baseline across participants design, relied on the use of a smartphone or tablet connected *via* Bluetooth to a two-switch device. This device served to select leisure and communication events and to control the smartphone or tablet’s delivery of step instructions for the activities scheduled. Data showed that during the baseline phase (with only the smartphone or tablet available), three participants failed in each of the areas (i.e., leisure, communication and functional activities) while one participant managed to access a few leisure events. During the intervention phase (with the support of the technology-aided program), all participants managed to independently access leisure events, make telephone calls, and carry out activities. These results suggest that the program might be a useful tool for helping people with intellectual and multiple disabilities improve their condition in basic areas of daily life.

## Introduction

People with mild to moderate intellectual disabilities or multiple disabilities are frequently reported to have serious difficulties in mastering independent access to leisure events, basic communication exchanges with partners who are not in their immediate environment, and performance of functional daily activities (Badia et al., 2013; Davies et al., 2018; Lancioni et al., 2018, 2020d; Lin et al., 2018; Desideri et al., 2021). Their difficulties with leisure events appear to be mainly related to their limited skills in operating devices commonly used to access those events (e.g., music devices, computers and tablets; Chan et al., 2013; Lancioni et al., 2018). Their difficulties regarding basic communication exchanges with distant partners appear to be linked to their limited skills in using telephone devices or comparable communication means (e.g., tablets and computers) essential for activating any of those exchanges (Lancioni et al., 2016; Darcy et al., 2017; Light et al., 2019). Finally, their difficulties with functional daily activities appear to be largely due to their failures in remembering all the steps of those activities and/or in performing those steps in the right sequence (Cannella-Malone and Schaefer, 2017; Goo et al., 2019; Desideri et al., 2021).

Given the vastly negative implications of those difficulties, the general consensus is that specific intervention programs need to be developed to address such difficulties (Boot et al., 2018). Programs developed for this purpose are to be capable of enabling people to become more independent in any or all of the aforementioned areas (leisure, communication, and daily activities) thus countering their dependence on staff or caregivers’ direct support/supervision. People’s reliance on external support/supervision, in fact, interferes with or prevents their development of initiative and self-determination and can prove quite expensive for staff and caregivers (Lancioni et al., 2020a,b; Wehmeyer, 2020; Wehmeyer et al., 2020). To increase the chances that the programs would succeed in reaching the goal, it might be critical to fit them with assistive technology solutions (Desmond et al., 2018; Lancioni et al., 2018; Light et al., 2019; Söderström et al., 2021).

In line with the above, a variety of efforts have been made to develop technology-aided programs that were focused on fostering independence in one of the aforementioned areas, that is, (a) leisure (Wang et al., 2011; Stasolla et al., 2015; Lancioni et al., 2016), (b) communication (van der Meer et al., 2012, 2017a,b; Kagohara et al., 2013; Ricci et al., 2017), or (c) performance of functional occupational/vocational activities (Mechling et al., 2010; Savage and Taber-Doughty, 2017; Desideri et al., 2021). The largely positive outcomes of those programs served as basis and incentive for the development of new programs that would target two of the areas or all three of them simultaneously. Programs targeting more areas increase the range of occupational opportunities available for the people involved and might thus represent a more functional/satisfactory option than programs focusing on a single area. Moreover, setting up programs that allow participants to alternate between various forms of occupation could be expected to motivate the participants to remain positively engaged for relatively long periods of time, thus increasing their overall level of independence and reducing staff and caregivers’ supervision costs (Kazdin, 2012; Lancioni et al., 2020c, 2022a).

Recently, programs were reported whose aim was to support people with intellectual and multiple disabilities in all three areas (i.e., leisure, communication, and daily activities; Lancioni et al., 2020e, 2022a). For example, Lancioni et al. (2022a) set up a program that relied on the use of (a) a smartphone with Android operating system, SIM card, Internet connection and Google account, and MacroDroid application, and (b) eight mini voice-recording devices, each containing a verbal message/request that could be activated by a hand pressure response. The messages/requests served to trigger the Google Assistant of the smartphone and thus to get the smartphone to deliver what the messages/requests indicated. The requests of four of the devices concerned preferred leisure options (e.g., songs). The requests of the other four devices concerned telephone calls or text messages to preferred communication partners. The program was arranged in such a way that periods of time with access to leisure and telephone calls were alternated with periods of time in which daily activities were to be carried out. During the latter periods, the smartphone provided the participants with instructions for the single activity steps. The results showed that the five participants managed to use the program and accessed leisure, communication, and daily activities independently.

These results can be taken as an encouragement to continue with the development of technology-aided programs aimed at supporting people with intellectual and multiple disabilities in the aforementioned areas. In developing new programs, one might focus on ensuring that they (a) require the use of a reduced number of technology devices and (b) allow the participants to control the instructions for the activities by themselves. A program that relies on a small number of technology components would be relatively easy to set up and to use across settings (e.g., compared to a program that involves a series of voice recording devices). A program, in which activity instructions are under participants’ control (rather than delivered at preset intervals), could help to ensure that instruction occurrences always coincide with participants’ readiness to respond. Obviously, pursuing the objective of instructional control may be justified when (a) participants are in the mild to moderate intellectual disability range, and therefore fairly likely to manage the instructions with low risk of omissions and confusions, and (b) the program includes the presentation of reminders in case the participants forget to activate the instructions (Lancioni et al., 2022b).

The present study was aimed at developing one such new program. The technology required by the program involved a smartphone or tablet connected *via* Bluetooth to a two-switch device. This device, which could be carried by the participants or placed in their proximity, was configured to enable them to select leisure and communication events and manage the delivery of step instructions for the activities scheduled during the sessions. Four participants with moderate intellectual disability and sensory and motor impairments were included in the study.

## Materials and methods

### Participants

Table 1 lists the four participants (three women and one man) by their pseudonyms and reports their chronological age, their sensory and motor impairments, and the age equivalents for their receptive and expressive communication as measured *via* the second edition of the Vineland Adaptive Behavior Scales (Sparrow et al., 2005; Balboni et al., 2016). The chronological age ranged from 28 (Camille) to 59 (Finley) years. The Vineland age equivalents on receptive and expressive communication varied between 4 years and 3 months and 5 years and 1 month, and between 3 years and 9 months and 4 years and 4 months, respectively. Communication occurred verbally for all participants. Given the bilateral hearing loss, Paul’s understanding in part relied on his ability to read the lip movements and facial expressions of his communication partners. Finley, Camille, and April were able to follow verbal instructions provided *via* smartphone or tablet. Paul, by contrast, had some difficulties with those instructions and preferred to use pictorial instructions instead. All participants attended rehabilitation and care centers. While no formal test scores were available, the psychological services of those centers had estimated the participants’ intellectual disability to be in the moderate range. That is, the participants were considered to be among a group of people that (a) includes about 10% of all the individuals with a diagnosis of intellectual disability, and (b) requires some assistance in different areas of daily life (Boat and Wu, 2015).

**Table 1:** Participants’ pseudonyms, chronological age, sensory and motor impairments, and Vineland age equivalents for Receptive communication (RC) and Expressive Communication (EC).

Participants (pseudonyms)	Chronological age (years)	Sensory and Motor Impairments	Vineland age equivalents ^1^^,^^2^
			RC	EC
Finley	59	Severe unilateral hearing loss, and absence of ambulation	4; 8	3; 9
Paul	46	Moderate to severe bilateral hearing loss, partially corrected through hearing aids, and absence of ambulation	5; 1	4; 2
Camille	28	Blindness	4; 3	3; 11
April	46	Moderate visual impairment, partially mitigated with the use of eyeglasses	5; 1	4; 4

1 The age equivalents are based on the Italian standardization of the Vineland scales (Balboni et al., 2016).

2 The Vineland age equivalents are reported in years (number before the semicolon) and months (number after the semicolon).

The participants were recruited for the study based on a number of criteria previously verified through direct observations and staff interviews. First, they were interested in accessing leisure events such as songs and videos/films and in making telephone contacts (audio or video calls) with preferred communication partners such as family and staff members. In spite of their interests, they typically relied on staff or caregivers’ support for leisure and communication *via* telephone. Second, they could carry out simple functional activities if provided with verbal or pictorial instructions related to the steps of those activities. Third, they had expressed willingness to use a technology system such as that adopted in the study (i.e., a system whose functioning had been shown to them) to independently access leisure and communication events and to carry out simple functional activities. Fourth, staff (a) were keen on the use of a technology-aided program for supporting the participants’ leisure, communication and activity engagement and (b) had approved the system set up for this study, which had been shown to them in advance.

### Ethical approval and informed consent

All participants (a) had been informed verbally and through demonstrations about the system used in this study (i.e., smartphone or tablet and Bluetooth switch device) and the way the system worked, and (b) had expressed their willingness to be involved in the study and use the system to access leisure, communication and functional activities. In light of the participants’ level of intellectual functioning, this willingness was deemed to be a reliable indication of their assent/consent to join the study. Nonetheless, in view of their inability to read and sign a consent form, their legal representatives were also contacted. Specifically, the legal representatives were asked to read and sign such form on the participants’ behalf. The study complied with the 1964 Helsinki declaration and its later amendments and was approved (including the aforementioned consent process) by an institutional Ethics Committee.

### Setting, research assistants, sessions, leisure and communication, and activities

The study was carried out within the rehabilitation and care facilities that the participants attended. The two research assistants in charge of the study (i.e., responsible for implementing the study sessions of all four participants and recording part of the data; see below) were psychology graduates, familiar with the use of technology-aided interventions with people with intellectual and multiple disabilities as well as with data collection procedures.

Baseline and intervention sessions were implemented on an individual basis, once or twice a day, 3 to 6 days a week (in accordance with the participants’ schedules). During baseline sessions, the participants were provided with a partially adapted smartphone or tablet (see below) and were invited by the research assistant in charge of the sessions to use such device to access leisure events and communication (e.g., songs and telephone calls). The research assistant also asked the participants to carry out functional activities. During the intervention sessions, the participants used the technology system developed for the study, which supported them in accessing leisure and communication and performing activities. For each activity, the system allowed the participants to control the verbal or pictorial instructions related to the single activity steps. Each session included four leisure and communication periods alternating with three activity periods (see below).

Eight or nine activities, which could include some common material/objects were available for each participant (e.g., placing different combinations of objects in bags or other containers, reordering a room, or restocking specific areas of the daily context). The activities, which varied across participants, included 12–20 (*M* = 15) steps. Some of the steps of the single activities could change across days so as to make the activities relevant for the participants and convenient for the context.

### Technology system

The technology system used during the intervention sessions involved a Samsung Galaxy smartphone (for Finley, Camille and April) or a Samsung Galaxy tablet (for Paul) combined with a Bluetooth Blue2 switch (i.e., a 16 × 7 × 2 cm device encompassing two adjacent pressure-sensitive buttons; AbleNet EAN: 186648000609) and a mini speaker. The smartphone and tablet were (a) equipped with a SIM card, (b) provided with Internet connection and Google account, and (c) fitted with the WhatsApp Messenger and MacroDroid applications. The MacroDroid served to program the smartphone or tablet’s functioning in line with the intervention conditions. The smartphone and tablet were also supplied with (a) a variety of audio and video files representing the participants’ preferred leisure events and (b) the communication partners’ telephone numbers and prerecorded answers to telephone calls (see below).

For Finley, Camille, and April, the pressure buttons of the Bluetooth Blue2 switch were identified through a smooth and a rough cover, respectively. At the start of the session, (a) the smartphone verbalized: “You can listen to music by pressing the smooth button or can call somebody by pressing the rough button.” If the participant pressed the smooth button, the smartphone verbalized (i.e., at intervals of 2–4 s) the names of four preferred singers (which could vary during the study). If the participant pressed the smooth button following a singer’s name, the smartphone played a song by that singer. If the participant pressed the rough button, the smartphone verbalized the names of four preferred communication partners (i.e., family or staff members which could vary during the study). If the participants pressed the rough button following one of the names, the smartphone set up a video call (Finley and April) or an audio call (Camille) with that partner. At least four songs (which could vary across sessions) were available for each singer. The songs were played for 1.5 min (see Lancioni et al., 2022a). With regard to the telephone calls, no time restrictions were available. If the partner did not answer the call, a prerecorded video message (Finley and April) or audio message (Camille) of that partner was automatically played by the smartphone. Following the end of a song or of a call, the smartphone would automatically repeat the phrase about the possibility of accessing music or making a call *via* the pressure buttons and the participant could make a new choice provided that no more than 3 min had elapsed from the start of that leisure and communication period.

If more than 3 min had elapsed, the smartphone invited the participant to carry out an activity (e.g., “Now you take a bag to start the activity”). The participant was to take a bag and then press one of the two pressure buttons to get the next instruction (e.g., “Put the toothpaste in the bag” or “Put the bag on the higher shelf of the cupboard”). After completing this step, the participant was again to press one button to obtain the next instruction. The system could also deliver reminders/encouragements in case the participants failed to seek a new instruction for a preset period of time. Following the last instruction for the activity, the smartphone verbalized again the phrase about the possibility of accessing music or making a call *via* the pressure buttons. The process continued as described above for the rest of the session, which contained four periods of leisure and communication choices alternating with three activities.

For Paul, the two pressure buttons of the Bluetooth Blue2 switch were covered with pictures illustrating leisure and video calls, respectively. At the start of the session, the tablet showed the image of those (leisure and communication) buttons on its screen as a signal that Paul could press one of the buttons to make his choice. If he pressed the button with leisure videos, the tablet showed, one at a time, four preferred video categories (e.g., dogs and comedy). If Paul pressed the leisure button in relation to an image/category, the tablet presented one of the four possible videos of that category. If Paul pressed the communication button, the tablet showed, one at a time, the photos of four preferred communication partners. Pressing the button in relation to one of those photos set up a video call with that partner. All other procedural conditions were comparable to those described for the other participants except that pictures were used instead of words.

### Experimental conditions and data analysis

The study was implemented according to a non-concurrent multiple baseline across participants design (Barlow et al., 2009). The baseline phase, which included different numbers of sessions for the participants (i.e., as required by the design), was followed by an intervention phase. The number of baseline sessions was preset for the different participants (with a minimum of five sessions required; see Lobo et al., 2017) and kept unaltered given that the participants did not show successful performance in any of the areas targeted in the study (i.e., leisure, communication, and activities) or marginally succeeded in one of those areas (see “Results”). A study coordinator, who had access to video recordings of baseline and intervention sessions, provided regular feedback to the research assistants about their performance (i.e., implementation of procedural conditions) during those sessions to ensure procedural fidelity (Sanetti and Collier-Meek, 2014).

The participants’ frequency of leisure events accessed and telephone calls made, and percentage of activity steps performed correctly were presented in graphic form. The data available for each measure were summarized over blocks of sessions. The differences between the baseline and intervention data of each participant on the single measures were analyzed using the percentage of non-overlapping data (PND) method (Parker et al., 2011). This method allows one to determine the size of the intervention impact by computing for each participant the percentage of intervention data points that exceed the highest baseline data point.

### Baseline

**Figure 1:**
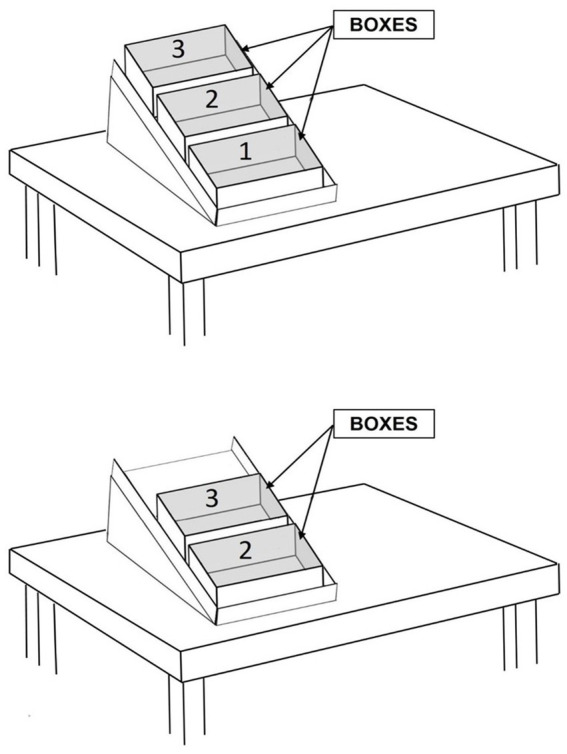
The upper section of the figure shows a desk with three boxes arranged on a sliding surface as used for Finley and Camille. Each box contained the material for one activity. The lower section of the figure shows how the second box would slide close to the participant once the first box was removed following the completion of the first activity.

The baseline included five to eight sessions. During those sessions, the participants sat (in a chair or wheelchair) in front of a desk where they found a smartphone or tablet, which had not yet been programmed *via* MacroDroid. The Bluetooth Blue2 switch was not available. The smartphone and tablet’s screen showed two folders. Clicking on (opening) the first folder allowed one to see four multimedia files. Clicking on one of the files led to the activation of a song or video. Clicking on the second folder allowed one to see the images of four preferred communication partners. Clicking on one of the partners led to the appearance of the telephone and video-call icons. Clicking on one of the icons started an audio or video call with the selected partner. At the beginning of a session, the research assistant verbally informed the participants that they could listen to songs or watch videos (a) by clicking on the first folder that she pointed out or (b) by uttering “Hey Google play singer’s NAME or video’s NAME on YouTube.” Subsequently, the research assistant told the participants that they could call their preferred partners (a) by clicking on the second folder that she pointed out or (b) by uttering “Hey Google call partner’s NAME.” Finally, the research assistant told the participants that there were three activities to be carried out. For Finley and Camille, the research assistant would then say, for example, “You should sort out the objects and put them into bags” while helping them point to or touch the materials to use (i.e., materials which were in boxes placed on the desk at which these participants sat; see upper section of Figure 1). For the other two participants, the research assistant could say: “You should rearrange the bathroom and the kitchen” and provide them with drawings of areas/objects to be used for the different activities. While talking to Paul, the research assistant ensured that he could see her face and understand what she told him (see “*Participants*”).

If the participants did not carry out any activity during the first 5 min of the session, the research assistant reminded them to do so. If the participant did not access any leisure (song or video) event and did not start any call within the first 7–8 min, the research assistant did it for them (i.e., started either a song/video or a call) so as to limit any sense of failure or frustration. If the participants did not carry out any activity or did not manage more than two steps per activity after 12 min, the research assistant provided a new reminder. The session would end after 2 or 3 additional minutes if the participants had not managed more than one new activity step. All the activity steps not carried out were rated as incorrect/omitted.

### Intervention

The intervention sessions differed from the baseline sessions in that the participants had the smartphone or tablet and Bluetooth Blue2 switch, which worked as described in the *Technology system* section. Finley and Camille had the smartphone and the switch on the desk where they sat and carried out their activities. April (who carried out her activities walking across different rooms) had the switch and a mini speaker fixed at her waist so she could seek activity instructions while moving in the rooms and could hear those instructions clearly through the mini speaker. The smartphone was on the desk where she sat during the leisure and communication periods. Paul (who carried out his activities moving across rooms with his wheelchair) had the switch and the tablet fixed to a container, which was secured to the wheelchair and served to transport the objects involved in the activities. In this way, Paul could activate the instructions and see them on the tablet’s screen while moving across different areas/rooms and could also spend his leisure and communication periods in any place he happened to be.

At the start of the sessions, the participants were allotted a 3-min leisure and communication period (see *Technology system*). Any leisure event or call started within the 3-min limit was to be completed regardless of the extra time it would add. At the end of this period, the participants were invited by the smartphone or tablet to start the first activity programmed for the session. The material for the activity was available on the desk at which Finley and Camille sat (i.e., in the box nearest to them; see upper section of Figure 1) or in two or three adjacent rooms including the one in which the session was started for April and Paul. The invitation to start the activity also included the instruction for the first response (i.e., for the first step of the activity) the participants were to perform. To receive any of the following step instructions, the participants were to press one of the switch buttons. In case they failed to press a button for a maximum preset time (determined by the research assistants for the different participants based on their performance speed), the smartphone or tablet would present a reminder/encouragement (i.e., verbally or through screen changes and vibration for Paul). The last instruction of the activity for Finley and Camille was to place the box (i.e., the first/nearest box from which they had taken the objects for the activity) to the side of their desk. Doing that caused the second/next box containing the material for the following activity to slide close to them (see lower section of Figure 1).

Following the last activity instruction, the smartphone or tablet informed the participants that they could again choose for music/videos and telephone calls using the switch buttons. The end of this second leisure and communication period led to a second activity, which was to be carried out as the first one described above. The session would end once the participants had completed four leisure and call periods and carried out three activities.

The first four to six sessions served as practice/introduction sessions. Initially, the research assistant used verbal and physical guidance to help the participants access leisure events, make telephone calls, and seek and respond to the activity instructions. Then, any form of guidance was faded out so that by the end of these sessions, all participants managed the use of the technology system successfully and accessed leisure events, made telephone calls, and started and carried out the activities independently. During the 57 to 85 regular intervention sessions that followed, no research assistant’s guidance was available except if the participant asked for help.

### Measures and data recording

The first three measures were: leisure events accessed, telephone calls made, and activity steps performed correctly (i.e., all were to be independent of research assistant’s guidance). The other two measures concerned smartphone or tablet’s encouragements and session duration. The smartphone/tablet automatically recorded all measures, except activity steps, during the intervention sessions. The research assistants in charge of the sessions recorded leisure events, telephone calls, and session duration during the baseline and activity steps performed correctly throughout the study. Interrater agreement was checked in all baseline sessions and at least 22% of the intervention sessions of each participant, by having a reliability observer join the research assistants in data recording. The percentage of interrater agreement on leisure and communication events and session duration during baseline (computed by dividing the number of sessions in which research assistant and reliability observer reported the same events and duration times differing less than 1.5 min by the total number of sessions, and multiplying by 100%) was 100% for all participants. The percentage of interrater agreement on activity steps performed correctly (computed for single sessions by dividing the number of steps with the same “correct” or “incorrect/omitted” score by the total number of steps and multiplying by 100%) was within the 90–100% range, with means greater than 98% for all participants.

## Results

**Figure 2:**
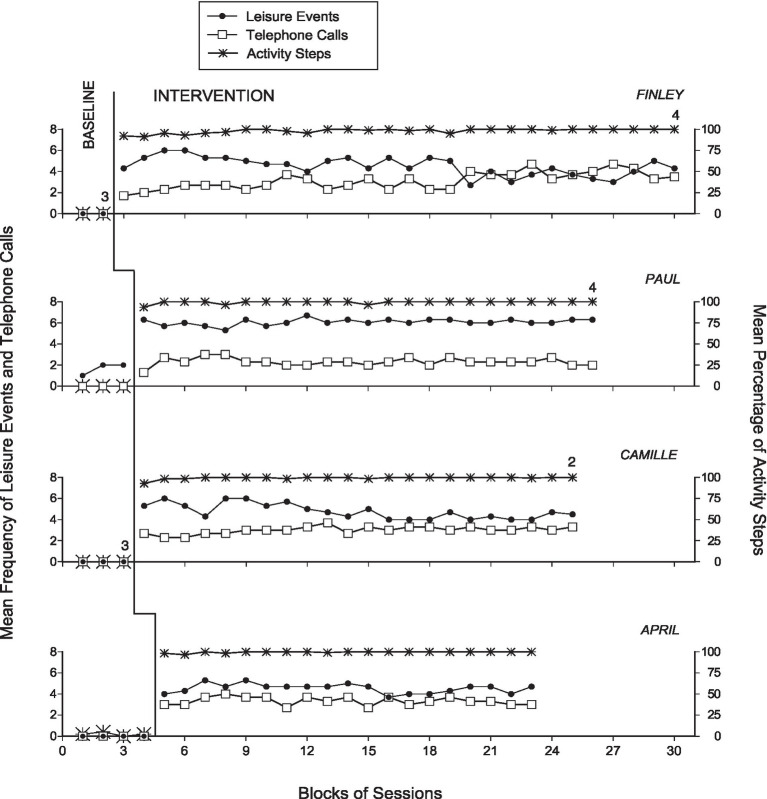
The four panels summarize the participants’ data during the baseline and intervention phases. The black circles and empty squares represent the mean frequency of leisure events accessed and of telephone calls made per session, over blocks of two sessions during the baseline and three sessions during the intervention. Blocks with different numbers of sessions (i.e., at the end of the phases) are marked with numerals indicating how many sessions the blocks include. The asterisks represent the mean percentage of activity steps carried out correctly over the same blocks of sessions.

The four panels of Figure 2 summarize the participants’ data during the baseline and intervention phases. The black circles and empty squares represent the mean frequency of leisure events accessed and of telephone calls made per session, over blocks of two sessions during the baseline and blocks of three sessions during the intervention phase. Blocks with different numbers of sessions (i.e., at the end of the phases) are marked with numerals, which indicate how many sessions those blocks include. The asterisks represent the mean percentage of activity steps carried out correctly over the same blocks of sessions. The panels do not report the practice/introduction sessions carried out at the start of the intervention phase.

During the five to eight baseline sessions, Finley and Camille did not manage to access leisure events, make telephone calls or carry out activity steps. Paul managed to access a mean of about 1.5 leisure events per session following many screen clicking attempts and repeated failures in activating the tablet due to his poor motor control. Yet, he did not make telephone calls and did not carry out activity steps. April failed to access leisure events and make telephone calls while carrying out a mean of about one correct activity step per session. The sessions were ended after 14 or 15 min due to lack of new correct activity steps following the second research assistant’s reminder.

The practice sessions introducing the participants to the intervention seemed to be very effective in helping the participants manage the use of the technology system (i.e., smartphone or tablet in combination with the Bluetooth Blue2 switch) successfully. This allowed them to be independent in their access to leisure events and telephone calls as well as in their performance of the activities scheduled for the sessions. During the 57 to 85 intervention sessions that followed the practice sessions, the participants were largely successful. Their mean frequency of leisure events accessed per session ranged between 4.5 (Finley and April) and 6.1 (Paul). Their mean frequency of telephone calls ranged between 2.3 (Paul) and 3.3 (April). These frequencies include both the telephone calls that were answered by the partners and those in which the participant received a prerecorded message of the partner called. Their percentage of correct activity steps was always above 98%, that is, errors/omissions were rare. Similarly, rare or totally absent were also the smartphone or tablet’s encouragements. This outcome was due to the fact that (a) the participants were consistent in their use of the switch device as well as in their performance of the activity steps and (b) the time intervals allotted to the participants for completing those steps and seeking new instructions (i.e., before an encouragement would occur) were apparently adequate. The mean session duration varied between about 24 min (Finley) and 41 min (Paul). Indeed, Paul required more time than the other participants for the performance of the activity steps due to his use of a wheelchair for moving across the activity rooms.

Comparisons between the baseline and the intervention data with the use of the PND method on leisure events, telephone calls, and activity steps carried out correctly showed indices of 1.0 (implying a strong intervention effect) for all participants. In fact, 100% of the intervention data points were above the highest baseline data point on each of those measures for all participants.

## Discussion

The data suggest that the technology-aided program used in this study was effective in enabling the participants to independently access leisure events, make telephone calls, and carry out daily activities. These results (a) corroborate the findings of previous studies aimed at helping participants with intellectual and multiple disabilities in those specific areas (Lancioni et al., 2020e, 2022a), thus supporting the applicability and potential of technology-aided programs in those areas, and (b) show the suitability of a relatively simple and portable technology system as the basis of a new intervention program. In view of the above, a few considerations may be put forward.

First, the participants’ consistent engagement in leisure and communication and their highly accurate performance of the activities scheduled for the sessions may have a number of explanations. For example, the program arrangement was most probably suited to the participants’ conditions and the technology system was easy for them to use (Federici and Scherer, 2017; Scherer, 2019). Accessing preferred leisure events and interacting/communicating with preferred partners in all probability represented forms of enjoyable engagement that motivated the participants’ initiative and performance continuity in these areas (Kazdin, 2012; Pierce and Cheney, 2017). It may also be added that the possibility of switching between these two areas could have played a positive role by allowing the participants to focus on what was more interesting and motivating for them at any specific time (King et al., 2014; Stasolla et al., 2015). Finally, the participants possessed the ability to carry out the steps of the activities scheduled during the sessions and the smartphone and tablet’s instructions seemed quite adequate to support the performance of those steps correctly. This combination probably gave the participants a sense of control and comfortableness (relevant to enhance motivation and personal satisfaction/mood) throughout the activity engagement time (Brown et al., 2013; Kocman and Weber, 2018).

Second, in this study, the prearranged smartphone or tablet’s encouragements were apparently unnecessary. In fact, the participants were consistent in activating the instructions and carrying out the related activity steps independently and thus did not really need this extra support from the technology system. However, a number of other participants with intellectual and multiple disabilities might have a lower level of performance consistency/continuity. A system that can be programmed to deliver reminders/encouragements in case of need (i.e., as the one used in this study) could be convenient to help these latter participants complete the activities and minimize errors (Kazdin, 2012; Pierce and Cheney, 2017).

Third, the technology system used in this study (a) relies on relatively few components/devices (i.e., a smartphone or tablet and a Bluetooth Blue2 switch) compared to systems used in previous studies (Lancioni et al., 2020e, 2022a) and (b) those components/devices are easily portable thus making the system practical for participants and staff. Indeed, in this study, one of the participants (Paul) carried both devices with him throughout the sessions. Another participant (April) had the Bluetooth Blue2 switch and a mini speaker fixed at her waist while the smartphone was at a desk where she sat to listen to music and make video calls. For the other two participants (Finley and Camille), who were sitting throughout the sessions, the devices were on the desk at which they sat. Yet, the fact that only two easily portable devices were involved made the staff’s task of setting up the sessions relatively simple.

Fourth, the system may be considered fairly accessible given that its components (smartphone or tablet and Bluetooth Blue2 switch) are commercially available (Boot et al., 2018; Desmond et al., 2018; Borg, 2019). Readily available is also the MacroDroid application used to program the functioning of the smartphone or tablet. Accessibility/availability does not however mean ready-made for use. In fact, the functioning of the smartphone or tablet needs to be programmed *via* the MacroDroid application before the beginning of the study. Programming requires some time and basic expertise on the part of the staff in charge of it. Another important aspect to take into consideration at this point is the cost of the technology. With regard to this aspect, it may be noted that the total amount can be around or slightly above US $500, which includes about US $200 or $250 for the smartphone or tablet and about US $250 for the Bluetooth Blue2 switch. The cost of the MacroDroid application is negligible. The cost of a mini speaker (to be used only for persons like April who move across different rooms for performing the activities and do not carry the smartphone or tablet with them) may be about US $25.

## Limitations and future research

Some basic limitations of the study may need to be pointed out here. The first limitation concerns the small number of participants involved. New studies would need to carry out direct and systematic replications of the present work to determine the strength and generality of the data reported as well as the possibility of introducing upgraded versions of the current technology system (Kazdin, 2011; Travers et al., 2016; Locey, 2020). A second limitation concerns the lack of generalization and maintenance assessment. In light of the reasons provided to explain the consistently positive performance of the participants throughout the intervention sessions, one might expect satisfactory maintenance results. Generalization might also be considered plausible given that the participants’ performance is relying on the technology system’s support (which remains unchanged) much more than on the context or people within it (which can change). Irrespective of the above, new studies would want to address these issues with proper generalization and follow-up evaluations (Pierce and Cheney, 2017).

A third limitation concerns the absence of an evaluation of participants’ satisfaction with the program. The data of the intervention phase seem to suggest that the participants were motivated to engage in the different areas addressed by the program and one may assume that motivation could hardly have existed in the absence of satisfaction (Kazdin, 2012; Stasolla et al., 2022). New studies might carry out an assessment of participants’ satisfaction by (a) asking the participants to choose between sessions with the support of the system (i.e., intervention sessions) and presumably positive alternative forms of daily engagement (Tullis et al., 2011), and (b) observing and comparing the participants’ behavior (e.g., expressions of positive mood such as smiles) during the two engagement situations (Dillon and Carr, 2007; Parsons et al., 2012).

A fourth limitation concerns the absence of a social validation of the program and the technology system on which it relies. Such validation could be arranged by interviewing staff personnel who are involved in the education and rehabilitation of individuals with intellectual and multiple disabilities. These personnel could be presented with short videos of the participants during standard intervention sessions and asked to provide their rating of those sessions in terms of content (practical relevance) and technology system used (Plackett et al., 2017; Worthen and Luiselli, 2019).

In conclusion, the results suggest that the technology-aided program was effective in helping people with intellectual and multiple disabilities to independently access leisure events, make telephone calls, and carry out daily activities. While the results are encouraging, one cannot make general statements about the program and the technology used for it until new research has addressed the limitations of this study. New research may also be directed at further developing the present technology system to improve its effectiveness and facilitate its use across participants with different needs.

## Data availability statement

The raw data supporting the conclusions of this article will be made available by the authors, without undue reservation.

## Ethics statement

The studies involving human participants were reviewed and approved by Ethics Committee of the Lega F. D’Oro (Osimo) Italy. The participants’ legal representatives provided written informed consent on behalf of the participants for their involvement in the study.

## Author contributions

GL was responsible for setting up the study, acquiring and analyzing the data, and writing the manuscript. NS, MO’R, and JS collaborated in setting up the study, analyzing the data, and writing/editing the manuscript. GA and AF contributed in working out the technological aspects of the study, acquiring and analyzing the data, and editing the manuscript. All authors contributed to the article and approved the submitted version.

## Conflict of interest

The authors declare that the research was conducted in the absence of any commercial or financial relationships that could be construed as a potential conflict of interest.

## Publisher’s note

All claims expressed in this article are solely those of the authors and do not necessarily represent those of their affiliated organizations, or those of the publisher, the editors and the reviewers. Any product that may be evaluated in this article, or claim that may be made by its manufacturer, is not guaranteed or endorsed by the publisher.
